# An Exploration of the Views of Teachers in a Dental College on the Current Performance Appraisal Framework and Promotion Policy

**DOI:** 10.7759/cureus.82270

**Published:** 2025-04-14

**Authors:** Vasanti Lagali-Jirge, Roopali Sankeshwari, Shivayogi Charantimath

**Affiliations:** 1 Oral Medicine and Radiology, Karnataka Lingayat Education Vishwanath Katti Institute of Dental Sciences and Hospital, Belagavi, IND; 2 Public Health Dentistry, Karnataka Lingayat Education Vishwanath Katti Institute of Dental Sciences and Hospital, Belagavi, IND

**Keywords:** academic career, academic recruitment, dental education, performance appraisal, promotion, teaching

## Abstract

Purpose

The current description of performance appraisal and advancement in the academic setting in India is nebulous, leading to confusion and a lack of preparation for promotions. The current system of promotions was introduced a few decades ago to meet the demand of a rapidly growing dental education sector. There was a shortage of teachers and having a time-based eligibility for promotion ensured that these vacancies were rapidly filled. This study was conducted to investigate the perceptions of teachers in dental colleges in India regarding the performance appraisal framework.

Methods

The sample comprised teachers who are currently or were previously employed as faculty in a dental college. We used a purposive sampling and snowballing method for recruiting participants. Teachers working in both public and private dental colleges throughout India were invited to participate in this survey. The self-designed questionnaire was validated and disseminated using Google Forms and WhatsApp. The results were analyzed using descriptive statistics and answers to open-ended questions were noted.

Results

There were 97 respondents to the questionnaire. All of them were engaged in multiple duties such as academic, administrative and clinical. All the respondents had a good number of publications. Around 45 (46.3%) teachers in the study sample got their first promotion on time. Sixteen (16.4%) of the respondents had applied for promotion through the Career Advancement Scheme (CAS), out of whom only 5% had been promoted. A majority of them (n=51; 52.5%) had up to five research papers where they were the first author whereas 23 (23.7%) of them had up to 10 papers as the first author during their academic careers. Eighty-six (88.6%) of the respondents shared that they fulfilled the eligibility criteria for a promotion. Twenty-seven (27.8%) teachers felt the current academic advancement framework was adequate for promotion. Fifty-five (56.7%) respondents felt the current promotion guidelines were not aligned with the goals and mission of dental education and academia, whereas 33 (34%) of them said the current promotion criteria were not congruent to the roles they were fulfilling in academia.

Conclusions

We feel that the current atmosphere in dental academia is one of general discontent towards performance appraisal and promotion policy as evidenced from the survey responses. Promotions are awarded to individual faculty members based on their accomplishments in research, teaching, and administrative responsibilities. Successful candidates must demonstrate that their accomplishments merit promotion. If we want to develop institutions as centers of excellence, we need to take a step back and reflect on where we are heading with the current framework for academic advancements, and then create a new one to achieve excellence.

## Introduction

The natural order of things is to keep progressing. In academics, teachers progressively attain higher posts through experience and accomplishments via promotions. This need for motivation, growth, and recognition has been established by Maslow’s hierarchy of needs [1]. A promotion is an advancement in the hierarchy at work and is a recognition of the individual's accomplishments at the workplace. A promotion is coveted because it brings with it a pay hike, benefits, and perks depending on the nature of the job, along with newer responsibilities [2]. It is important for any organization to evaluate an employee’s performance contiguously to ascertain whether they continually demonstrate competence in their assigned roles and responsibilities [3]. This is particularly true in healthcare institutions and higher education. The processes involved in academic performance appraisal should evolve to reflect the dynamic nature of education. The current description of performance appraisal and advancement in the academic setting in India is nebulous, leading to confusion and a lack of preparation for promotions.

Historical context of academic advancement: the need for change

Dental colleges in India are either publicly or privately funded. The number of dental colleges has grown by 215.9% in just two decades [4]. There are currently 6,942 seats for postgraduate training in India [5]. This sudden rise in the number of colleges and graduates created a demand for teachers [6]. The current system of promotion was introduced a few decades ago to meet the demand of a rapidly growing dental education sector. There was a shortage of teachers and introducing the time-based eligibility for promotion ensured that these vacancies were rapidly filled. As the number of dental colleges increased, the number of graduates with a Master of Dental Surgery (MDS) degree also increased, resulting in a rapid saturation of positions and the concentration of teachers in each rank. In the past, promotions had primarily been awarded to fill vacancies; however, this approach no longer appears to be viable. This is because dental colleges now have a diverse talent pool and teachers must demonstrate proficiency in multiple roles. The current academic ranks in a dental college are lecturer, reader (or associate professor), and professor. The current promotion policy, by design, has established a minimum requirement to be eligible for promotion. These include the number of years in service and a point system for publications in journals of varying latitudes. Post-graduate teachers are required to meet a minimum publication requirement, which is reviewed every three years [7]. 

There is a lack of clarity and objectivity on the type of scholarly activity to be pursued to be eligible for a promotion. Traditional metrics of scholarly activity include teaching, research, conference presentations, and research papers [5]. Currently, the Dental Council of India (DCI) has not clearly defined the competencies of an entry level academic faculty member. The only requirement is an MDS degree. During this degree, acquiring clinical/ practical skills and research remain the areas of focus. However, as a teacher, one steps into much larger shoes and aims to fulfill multiple roles which are mostly unclear before entering academia. There is a veneer of uniformity in the form of administrative, teaching, research and publications, and extramural activities. However, each university has modified these definitions to meet contemporary demands. Due to the pooling and saturation of teachers in senior positions in dental colleges, it has become increasingly difficult for aspiring and competent junior teachers to obtain promotions through the traditional performance appraisal frameworks. Many universities in Western and other developed countries practice the tenure and non-tenure track for promotion [5]. Career advancement takes place on both tracks. However, the requirements for each are different [8]. Further discussion on non-Indian systems of promotion is beyond the scope of this article.

Our study hypothesized that there is a general discontent among Indian dental teachers about the current promotion policy. This survey was undertaken to explore the perceptions of teachers about current practices in performance appraisal and promotions. The objectives of the study were to explore the views of dental teachers regarding performance appraisal and their experience with the promotion process. The primary outcome was to gather the views of the faculty on the current promotion policy in the dental colleges. 

## Materials and methods

This survey was conducted via a questionnaire after obtaining institutional ethical clearance (institutional review board (IRB) no. EC/NEWINST/2021/2345/1627). This study was conducted over two months from January to February 2023 at the Karnataka Lingayat Education Vishwanath Katti Institute of Dental Sciences and Hospital, Belagavi, India. The sample comprised teachers who are currently or were previously employed as faculty in a dental college. We used a purposive sampling and snowballing method for recruiting participants. Informed consent was taken via the questionnaire. Teachers working in both public and private dental colleges throughout India were invited to participate in this survey. The self-designed questionnaire was validated and disseminated using Google Forms (Google, California, US) and WhatsApp (Meta, California, US). Data was saved in MS Excel (Microsoft Corp., Redmond, WA, US) and used for analysis. The results were analyzed using descriptive statistics, and answers to open-ended questions were noted. 

Questionnaire validation

A self-designed questionnaire with 31 questions was developed after consulting subject experts. During the first stage, the study questionnaire was prepared in English and checked for comprehensiveness. A few questions were then modified, and the final questionnaire consisted of 27 questions. The survey included questions on the respondent's current demographic and work profile (questions one to seven), awarding of promotions (questions eight to 13), number of publications (question 14), and perceptions about the current performance appraisal and promotion policy (questions 15 to 27). There were 21 closed-ended questions and six open-ended ones. During the second stage, the final approved questionnaire was shared with 15 participants. After a month, the same questionnaire was shared once more with the same participants to verify the reliability of the answers. A Cronbach’s alpha of 0.80 was obtained, reflecting a high degree of reliability. The sample size was estimated using the formula n = z2pq/d2, where p= 50%, q=50%, d=10. Based on this formula, the sample size for the study was 100.

## Results

There were a total of 97 respondents to the questionnaire and the survey also obtained their consent. Their demographic profile is presented in Table 1. Their experience in academia ranged from zero to more than 15 years.

**Table 1:** Demographic profile of the study participants

Demographic profile	Number	Percentage
Gender		
Male	43	44.33
Female	54	55.67
Designation/rank		
Lecturer	28	28.87
Reader	40	41.24
Professor	22	22.68
Head	7	7.22
Qualifications		
Master of Dental Surgery (MDS)	97	100.00
Post Graduate Diploma in Health Professions Education (PGDHPE)	10	10.31
Ph.D.	11	11.34
Foundation for Advancement of International Medical Education and Research (FAIMER) Fellowship	4	4.12
Post-graduate (PG) Diploma	2	2.06
Other	7	7.22
Current academic workplace		
Private	89	91.75
Government	2	2.06
Not in college	6	6.19
Total	97	100.00
Academic experience		
Years in academia		
0-5	15	15.46
6-10	22	22.68
11-15	33	34.02
>15	27	27.84
Years in your current designation		
0-5	47	48.45
6-10	32	32.99
>10	18	18.56
Total	97	100.00

All the respondents were engaged in multiple teaching roles such as teaching undergraduates (UG), postgraduates (PG), guiding PhD students, administrative responsibilities, member of internal quality assurance committees (National Assessment and Accreditation Council (NAAC) and National Accreditation Board for Hospitals & Healthcare Providers (NABH)), research, clinical duties, conducting continuing dental education (CDE) programs/workshops, organizing conferences, administrative roles in university, and student welfare activities (Table 2).

**Table 2:** Current roles and faculty perception on criteria for awarding promotions NAAC: National Assessment and Accreditation Council; NABH: National Accreditation Board for Hospitals; CDE: Continuing Dental Education

Roles fulfilled/performed in the current workplace	Number	Percentage	Criteria for awarding promotion	Total	Percentage
Teaching undergraduates (UG)	97	100.00	Research	85	87.63
Teaching postgraduates (PG)	76	78.35	Conference presentation	65	67.01
PhD guide	8	8.25	Publications	78	80.41
Administrative	36	37.11	Book authorship	48	49.48
NAAC duties	67	69.07	Grants	43	44.33
NABH duties	20	20.62	Teaching	94	96.91
Research	68	70.10	Student mentoring	80	82.47
Clinical duties	80	82.47	Administrative responsibilities	46	47.42
Conducting CDE programs/ workshops	59	60.82	Conduct	81	83.51
Organizing conferences	41	42.27	Patents	35	36.08
Internal Quality Assurance Committee	31	31.96	Continuing Professional Development	40	41.24
Admin roles in university	13	13.40	Administrative committees	68	70.10
Student welfare activities	44	45.36	Invited guest lectures	55	56.70
Student admission	13	13.40	Community Service	57	58.76

With reference to receiving promotions (Table 3), we asked the participants if they received their promotions on time.

**Table 3:** Teachers' experiences and perceptions of the promotion framework

Items	Number	Percentage
Did you receive your first promotion on time?		
Yes	45	46.3
No	52	53.6
Did you receive your second promotion on time?		
Yes	23	23.71
No	74	76.29
Have you applied for promotion through the career advancement scheme (CAS)?		
Yes	16	16.49
No	81	83.51
How many original research papers have you published as a first author since your first day as faculty?		
0–5	51	52.58
6–10	23	23.71
11–15	8	8.25
>16	15	15.46
Have you fulfilled the requirements for eligibility to the next promotion?		
Yes	86	88.66
No	11	11.34
Do you feel the current framework for academic advancement (promotion) is adequate?		
Yes	27	27.84
No	70	72.16
Do you feel the current promotion guidelines are in alignment with the goals and mission of dental education and academia?		
Yes	7	7.22
No	55	56.70
NA	35	36.08
Do you feel that current promotion criteria are congruent to the roles you are fulfilling in academia?		
Yes	33	34.02
No	64	65.98
Do you feel the current roles and responsibilities performed by you are considered important in promotion interviews/appraisals?		
Yes	53	54.64
No	44	45.36
Do you feel promotions should only be given against vacancy?		
Yes	12	12.37
No	85	87.63
Do you feel all teachers need a mentor/coach to perform well and give you honest feedback on your performance as a faculty member in a dental college?		
Yes	57	58.76
No	12	12.37
I don't know	28	28.87
Do you feel performance expectations can be generalized across different dental departments?		
Yes	47	48.45
No	50	51.55
Total	97	100.00

Around 46.3% and 23.7% of the study sample got their first and second promotions on time, respectively. A few of them (16.49%) secured promotions through the career advancement scheme (CAS). All the respondents had published research papers. A majority of respondents felt that the current framework for academic advancement is inadequate (72.16%) and the current promotion guidelines do not align with the goals of dental education and academia (56.70%).

Among those who did not receive their promotions on time, a majority of them reported no vacancy/requirement according to the DCI norms as the reason. Other reasons mentioned were ‘lack of funds in the college’, ‘delays by college administration’, ‘managements do not want to promote the teachers because they will have to increase the salaries’, ‘pooling of teachers who are awaiting promotion.’ About 16.49% of the respondents had applied for promotion through the CAS and all the participants had a good publication record.

Nearly 88.6% of the respondents said they had fulfilled the eligibility criteria for a promotion. On being asked about the most important requirements for a promotion in their institution/university, the responses included ‘availability of clear vacancy’, ‘the prerequisite years of experience’, ‘management decisions or readiness’, ‘publications’, ‘research grants’, 'PhD’, ‘having connections with the management or the right connections’, and ‘having a portfolio that includes research and participation in university activities.’ Some respondents mentioned that the guidelines were not clear or they were not aware of the expectations. One of the participants mentioned that the promotion was not awarded although all the criteria were fulfilled. Another highlighted the importance of academic knowledge and teaching skills.

About 27% of the participants felt that the current academic advancement framework was adequate for promotion. On the other hand, 56% of them felt that the current promotion guidelines were not aligned with the goals and mission of dental education and academia, and 34% stated that the current promotion criteria were not congruent with the roles they were fulfilling in academia. Around 56% of the study participants agreed that their current roles and responsibilities were considered important in promotion interviews/appraisals, whereas 13% stated that a promotion should be given only when there was a clear vacancy (Table 3). When asked about their awareness of the promotion criteria for health professional faculty in other parts of the world, most respondents seemed unaware. A few reported the use of a point system based on criteria such as work output, experience and participation in research, credit-based systems, and patient satisfaction.

Some responses in this regard are given below:

"Weighted scores self-assessment, peer assessment, and superior assessment taken together by weightages.”

“Yes. Worldwide they are more elaborate and do not rely only on the number of years spent in a dental college. Other aspects like continuing education, invited lectures, and keynote lectures are given importance.”

A majority of them (58%) said that all teachers need a mentor/coach to perform well and give honest feedback on their performance as a faculty member. Almost 47% of the participants felt that performance expectations can be generalized across different dental departments (Table 3). When asked about what should be included in the performance appraisal framework for academicians, participants highlighted factors such as the availability of vacancies, the faculty member’s experience, and their portfolio, including research contributions, involvement in university activities, management readiness or decisions, publications, research grants, and PhD supervision. Some respondents stated that promotion was awarded based on DCI norms, whereas others stated that closeness/connections or sway with the management mattered.

We then asked the participants about the barriers to reforming the promotion guidelines. They opined that the regulatory or higher authorities lacked vision, managements developed their own rules, and there was a lack of appreciation in teaching and clinical work. Other barriers included more focus on publications at the expense of patient care, lack of authentic research, promotion based on availability of vacancy, and financial burden on colleges due to pay hike associated with the promotion. Some participants also noted the presence of discrimination and internal politics affecting promotions.

The final open-ended question in the survey sought to understand the respondents' perspectives on making the academic advancement framework more objective and robust. Participants suggested a range of measures beyond just experience and publications, including regular appraisals, structured training programs, 360-degree assessments, strong leadership, and a robust DCI evaluation process. They also emphasized the importance of anonymous reporting mechanisms, performance-based promotions, a transparent credit system, mentoring and support for competent teachers, a merit-based point system, and standardized teacher training across institutions. 

## Discussion

This study aimed to explore the perceptions of dental college teachers regarding performance appraisals and career advancements. The results offered valuable insights into various faculty-related aspects of dental education in India, highlighting key implications such as the need for mentorship, effective performance appraisal systems, addressing barriers to reform, and strengthening the objectivity and rigor of academic advancement frameworks in dental colleges. 

Literature on the views and opinions of the faculty regarding performance appraisals and promotions in dental colleges is scarce. The results of our survey are similar to the opinions expressed by Costello et al. [8]. The respondents included lecturers, readers, professors, and heads of departments. A higher percentage of respondents work in private dental colleges. Private institutions provide the majority of dental education in India. There are 279 colleges offering postgraduate training 323 colleges offering undergraduate training, and 20,844 specialists employed in these colleges as teachers. Approximately 14% of these are government-owned colleges and the rest are privately owned [5]. This may play a role in resource availability, funding, and management decisions, affecting promotions and other aspects of faculty development. Despite possessing advanced qualifications, some teachers have not received promotions on time. The presence of advanced qualifications does not appear to be a contributing factor in these promotions as respondents with a PhD also have reported delays.

**Figure 1:**
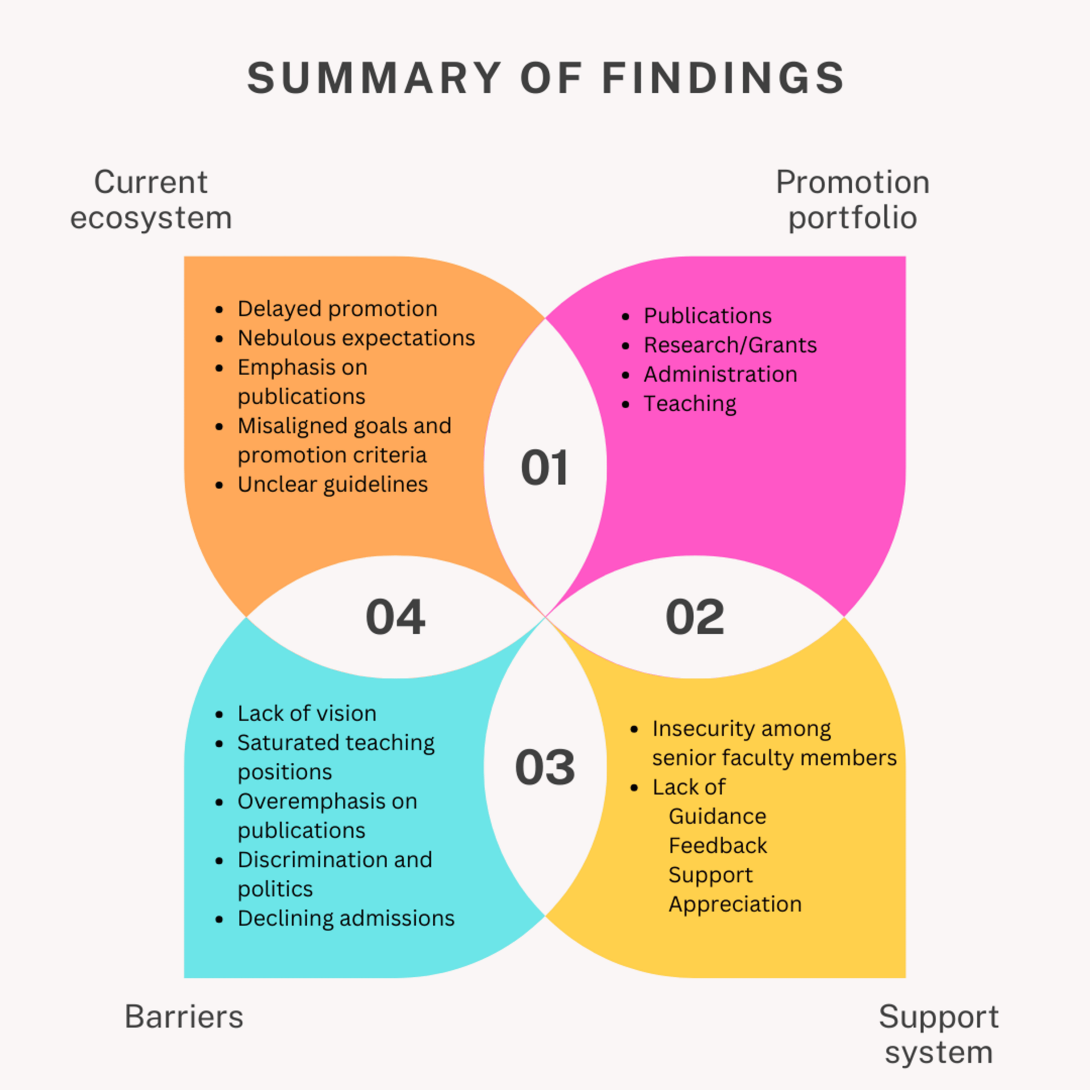
Summary of findings Image created by Dr. Jirge on Canva (Perth, Australia)

The respondents to this survey represented a broad spectrum of professional experience. We found that there was a higher percentage of respondents with more than 15 years of experience who had been serving in their roles for an extended period. The multifaceted roles fulfilled by the faculty members included teaching, research, clinical duties, administrative responsibilities, and participation in various institutional activities. We organized the discussion under four important themes (Figure 1).

Current ecosystem

Many respondents expressed a lack of alignment between promotion guidelines, the goals of dental education, and their roles in academia. This reflects the need for a more inclusive promotion framework. Some respondents mentioned that candidates who have connections with the management are favored during promotions. Such detrimental practices can derail careers and lead to disillusionment of truly deserving teachers. Promotion policies may vary through geographic regions based on local frameworks. While many emphasize research and publication, some countries report regulations that are in favor of administrative functions [9]. Other studies report a lack of transparency, absence of specialized staff in promotion committees, long processes, and unnecessary administrative requirements [9]. We observed a limited awareness among respondents about the criteria for promotion in other parts of the world for health professions faculty. Exploring and learning from promotion systems in other countries could provide valuable insights for improving the existing promotion framework. Many universities overseas offer promotions in the tenure and non-tenure tracks. These are highly competitive and establish high standards in academia [5].

Portfolio for promotions

A clearly articulated promotion framework allows teachers to prepare for appraisals and promotion. It also has the potential to make the selection process fair. The promotion policies vary between government and private dental colleges. The responses on the components of a performance appraisal framework for academicians suggest that diverse factors should be considered. These include the presence of vacancies, experience, research portfolio, participation in university activities, management decisions and readiness, publications, research grants, and PhD qualifications. However, it is worth noting that some respondents mentioned unclear guidelines or a lack of awareness of expectations, which calls for transparent and well-communicated performance evaluation criteria and clearly articulated job descriptions.

The suggestions provided by the respondents on how to make the academic advancement framework more objective and robust offer valuable insights. An exploration of perceptions about promotion guidelines in a medical college has found that promotion guidelines should be streamlined through a robust promotion policy and should include comprehensive teacher assessment with mandatory continuing professional development activities and a standardized approach to research work [10]. Most of the respondents seemed unaware of the promotion policies in dental colleges in other countries. At the global level, a majority of the universities in developed nations practice the review, tenure, and promotion system which has its own set of advantages and challenges [3,8]. The promotion guidelines should be consistent with the activities faculty members are asked to perform to fulfill the institutional mission [8].

Support system

Teachers are the lifeline of an educational institution and therefore need support, nurturing, and mentoring [3]. The majority of respondents emphasized the importance of mentorship, coaching, and performance feedback in enhancing teaching effectiveness. This highlights the need for structured mentorship programs within dental colleges to support the professional development and growth of faculty members. Mentorship can provide guidance, support, and constructive feedback, enabling faculty members to enhance their teaching and academic skills, and it is integral for personal growth and professional development [4,11]. Faculty mentoring programs offer an undeniable advantage to a teacher [12]. There is scarcity of literature on mentoring within the dental faculty, though few dental schools in the USA offer formal or informal mentoring programs for teachers. Similarly, literature on mentoring of dental teachers in India appears to be scarce. However, mentoring of physicians [13] and nursing faculty [14] appears to be widely practiced. Mentorship is known to have a significant influence on different facets of professional life including personal development, career guidance, specialty/career choice, faculty retention, and research productivity, including publication and grant success [15]. Navigating the promotion scene becomes competitive with each new generation of teachers. They are expected to perform multiple roles like teaching and patient care along with research and administrative duties. Therefore, they would require mentoring early in their careers.

Barriers to reform

The opinions shared by the participants underscore the key challenges in revising the promotion guidelines. These include a perceived lack of vision among regulatory or higher authorities, the development of arbitrary rules by college managements, insufficient recognition of teaching and clinical work, overemphasis on publications at the expense of patient care, absence of authentic research, financial burdens associated with promotions, and issues of discrimination and politics in promotion decisions. Addressing these barriers is crucial to ensure fair and effective reforms in the promotion guidelines. A survey by Samuel et al. [16] has found that a delay in promotions or a lack of transparency in the process is usually followed by a general sense of deprivement and a loss of motivation to work, thereby affecting the quality of teaching and academia. Similar observations have been found in an online survey by Diamantes [17].

Overall, the implications of these results underscore the complexity of the promotion process in Indian dental education and academia. Addressing these factors could contribute to the professional growth and development of teachers, ultimately enhancing the quality of dental education and research. Scholarship and research have been key metrics for determining a teacher’s eligibility for a promotion. With increased responsibilities in educational institutions, other roles also need to be considered in awarding a promotion and associated incentives. The expectations for promotion and the policies thereof have to be made known. The CAS was introduced by some Indian universities as an opportunity for eligible teachers to apply for promotion without vacancy, though we do not have supporting information for this. The University Grants Commission (UGC) uses the CAS in lieu of promotion [18].

As evidenced from the survey, the current atmosphere in dental academia is one of general discontent towards performance appraisal and promotion policy. The goal of any educational institution is to create professionals for tomorrow. This is ensured by scholarship and scholarly activities. Boyer’s model of scholarship, introduced in 1990, outlines the four key dimensions of discovery, integration, application, and teaching, which provide a valuable conceptual framework for performance reviews in academic institutions [19]. To foster a culture of continued excellence and align with global standards, academic institutions, as centers of excellence, should embed these dimensions of scholarship into their academic culture. In line with this, Janjua et al. have recommended a comprehensive performance review system that includes mandatory professional development activities and evidence-based approaches to promotions [10].

**Figure 2:**
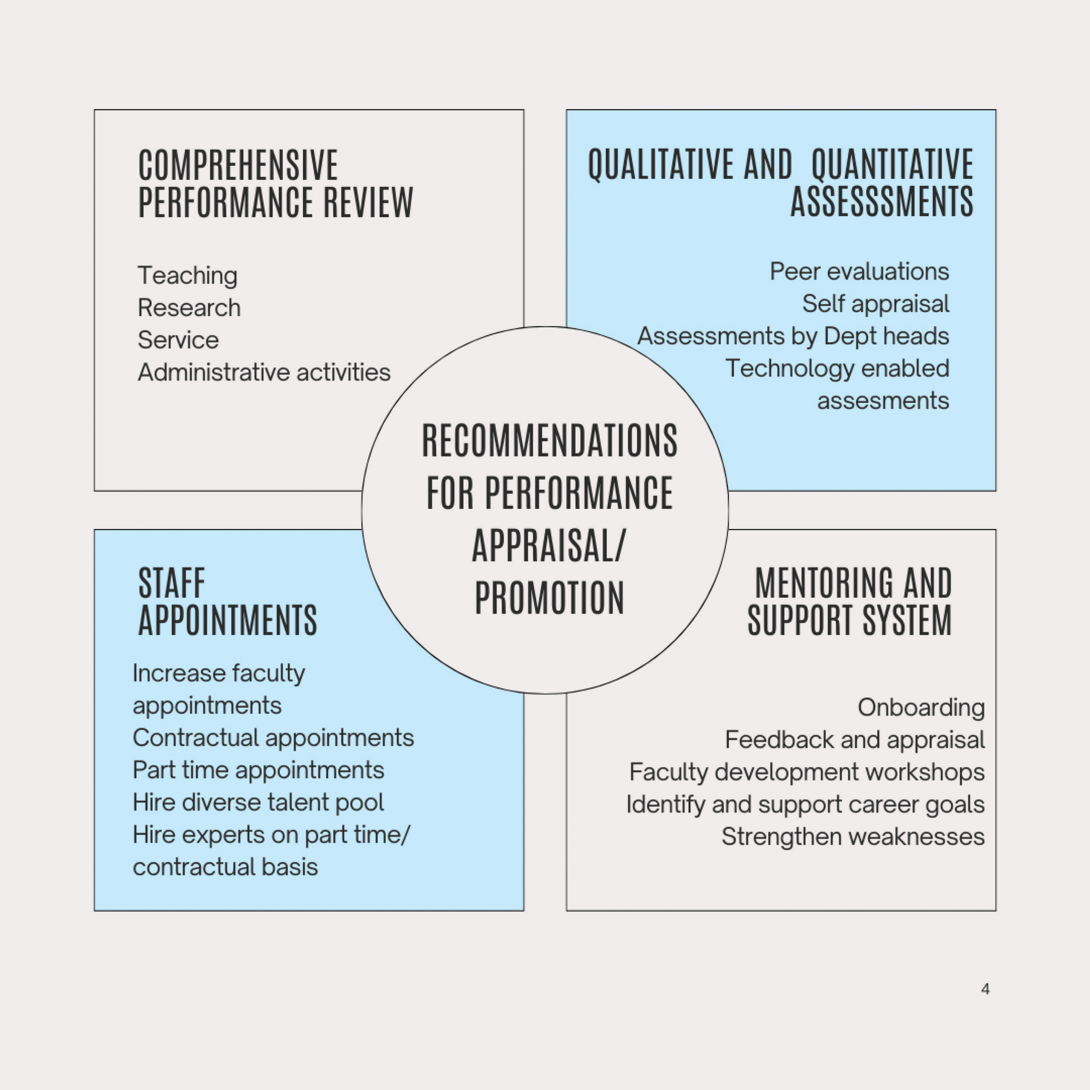
Recommendations for performance appraisal and promotion of teachers in dental colleges Image created by Dr. Jirge on Canva (Perth, Australia)

We feel that the current prescriptive system of basing promotions on the number of years of experience along with points for publication is obsolete for a number of reasons. First, the experience system was necessary at a time when there was a scarcity of teachers. Second, the roles of a teacher have diversified. Teachers in dental colleges are performing multiple tasks and scholarly activities. We have a surplus of qualified professionals who want to pursue a career in academia, and therefore, need to provide clearer guidelines. Third, we need a robust framework to ensure a fair opportunity to all contenders. A further study of this matter is required with a pan-Indian sample. Based on the findings of this survey and literature sources, we have made a few recommendations (Figure 2). Since these recommendations may not meet the requirements for an overhaul, a Delphi approach with multiple stakeholders is required to bring in reforms in the promotion process. 

Recommendations for performance appraisal and promotions

1. Conduct a comprehensive performance review, which includes teaching, research, service, and administrative activities. Absolute and relative standards can be used to perform reviews [18] along with transparency in the process of awarding or declining promotions.

2. Use quantitative and qualitative methods of peer evaluations, self-appraisal, assessments by department heads, and technology-enabled assessments via performance management systems [19].

3. Develop mentoring and support systems for employee wellbeing using onboarding, feedback and appraisal, faculty development workshops, to identify and support career goals while strengthening weaknesses.

4. Ensure overhauling of the staff appointment guidelines by the apex bodies. Increase faculty appointments, include contractual appointments, part time appointments, and hire diverse talent pool of experts in focused areas on part time or contractual basis.

Reflective critique

We have not conducted an in-depth exploration of the relationship of gender and ethnicity on performance appraisal. We have received responses from teachers with experience ranging from as little as a few months to over 15 years. The respondents in our study came from government and private dental colleges, and were affiliated to state universities or autonomous institutions. As such there will be bias due to a large variation in the baseline characteristics. Hence, the outcome of this variation is beyond our control. A significant proportion of the study's respondents were in senior positions (e.g., professors and department heads). This may lead to a response bias because they might view delayed promotions differently from the teachers who aren’t promoted. Focus group discussions and detailed interviews can provide a deeper understanding from the different ranks of teachers. The results may not be generalizable because the sample size was small and inferential statistical analyses were not done. While conducting the survey through Google Forms, we encountered challenges in securing responses. We believe this issue can be addressed by including a personalized note with the survey and sending follow-up reminders. Although open-ended questions were used, a thematic analysis of the responses was not carried out.

## Conclusions

In our study, we observed that faculty promotions are typically based on individual achievements in research, teaching, and administrative responsibilities. While candidates are expected to demonstrate that their accomplishments warrant promotion, the processes involved can often be complex and difficult to navigate. In recent years, faculty members have increasingly taken on diverse roles, with no two individuals performing the same functions. This evolving landscape calls for a revision of existing performance appraisal and promotion policies. A modern, robust promotion framework must acknowledge the full spectrum of academic contributions, not just research publications. Moreover, transparency and fairness in evaluating these multifaceted roles are essential.

At the same time, it is critical to preserve the core values that underpin academic institutions. To build them as true centers of excellence, we must critically examine our current frameworks for academic advancement and create a new framework that reflects the complexity of the work performed by the faculty. Drawing insights from promotion policies in developed countries can provide a strong foundation for building such an actionable framework.

## Appendices

Questionnaire

Q1.  Gender

        a.      Female

        b.      Male

Q2.  What is your highest qualification?  Select all that apply.

        a.      BDS

        b.      MDS

        c.      PGDHPE

        d.      FAIMER

        e.      PhD

        f.        Other

Q3.  Current designation/rank

        a.      Lecturer/ Assistant Professor

        b.     Reader/ Associate Professor

        c.      Professor

        d.      Head

Q4.  Which of the following describes your current academic workplace?

        a.      Private

        b.     Government

        c.      I don’t work in a college.

Q5.  What are the roles you are fulfilling/or have performed in your current workplace?

        a.      Teaching UG

        b.      Teaching PG

        c.      PhD guide

        d.     Administrative

         e.     NAAC

         f.     NABH

         g.    Research

         h.    Clinical duties

         i.     Conducting CDE programs/ workshops

         j.     Organising conferences

         k.    IQAC

         l.     Admin roles in university (if you are an autonomous institution)

         m.   Student welfare activities

         n.    Student Admission

Q6.   How many years have you been in academia?

         a.    0 - 5

         b.    6 - 10

         c.    11 - 15

         d.    >15

Q7.   How many years have you served in the current designation?

         a.     0 - 5

         b.     6 - 10

          c.    >10

Q8.   Did you receive your first promotion on time?

         a.     Yes  

         b.     No

Q9.   If no, what was the reason? (open ended)

Q10. Did you receive your second promotion on time?

         a.     Yes

           b.   No

Q11.   If no, what was the reason? (open ended)

Q12.   Have you applied for promotion through Career advancement scheme (CAS)?

           a.   Yes

           b.   No

Q13.   If yes, did you get the promotion?

           a.   Yes

           b.   No

Q14.   How many research publications have you published in as a first author in since your first day as a faculty?

           a.   0 - 5

           b.   6 - 10

           c.   11- 15

           d.   >15

Q15.   What are the most important requirements for promotion in your institution/ University? (open-ended)

Q16.   Have you fulfilled the requirements for eligibility to the next promotion?

           a.   Yes 

           b.   No

Q17.  Do you feel the current framework for academic advancement (promotion) is adequate?

           a.   Yes

           b.   No

Q18.   Do you feel the current promotion guidelines are in alignment with the goals and mission of dental education and academia?

           a.   Yes

           b.   No

Q19.   Do you feel that current promotion criteria are congruent to the roles you are fulfilling in academia?

           a.  Yes

           b.   No

Q20.   Do you feel the current roles and responsibilities performed by you are considered important in promotion interviews/ appraisals?

           a.   Yes

           b.   No

Q21.   Do you feel promotions should only be given against vacancy?

           a.  Yes

           b.  No

Q22.   Are you aware of promotion criteria for health professions faculty in other parts of the world? If yes, please elaborate? (open ended)

Q23.   Do you feel all teachers need a mentor/coach to perform well and give you honest feedback on your performance as a faculty member in a dental college?

           a.  Yes

           b.   No

           c.   I don't know

Q24.   Do you feel performance expectations can be generalized across different dental depts?

           a.   Yes

           b.   No

Q25.   What in your opinion should be included in performance appraisal framework of academicians? (Select all that you feel are relevant)

            a.  Research

            b.  Conference presentation

            c.  Publications

            d.  Book Authorship

            e.  Grants

            f.  Teaching

            g.  Student Mentoring

            h.  Administrative responsibilities

            i.   Conduct/ Misconduct

            j.  Patents

            k. Continuing Professional Development

            l.  Administrative committees

           m. Invited guest lectures

           n.  Community Service

Q26.   What in your opinion are barriers to reform in promotion guidelines? (open-ended)

Q27.   What in your opinion needs to be done to make academic advancement framework objective/robust? (open-ended)
